# Uterine leiomyoma in a 13-year-old adolescent successfully treated with laparoscopic myomectomy: A case report

**DOI:** 10.1097/MD.0000000000018301

**Published:** 2019-12-10

**Authors:** Natsuko Morita, Tomohito Tanaka, Sosuke Hashida, Satoshi Tsunetoh, Kohei Taniguchi, Kazumasa Komura, Masahide Ohmichi

**Affiliations:** a Department of Obstetrics and Gynecology; b Translational Research Program, Osaka Medical College, Takatsuki, Japan.

**Keywords:** juvenile, laparoscopic surgery, uterine leiomyoma

## Abstract

**Rationale:**

Uterine leiomyoma, which is very common gynecological tumor in the reproductive years, is extremely rare in adolescence. We herein report a case of a uterine leiomyoma treated with laparoscopic surgery in an adolescent.

**Patient concerns:**

A 13-year-old girl with no gravida and her first menses at 11 years of age reported feeling bloated. She had a regular menstrual cycle but felt increased abdominal distension.

**Diagnosis:**

Transabdominal ultrasound and magnetic resonance imaging revealed uterine leiomyoma with a diameter of 10 cm.

**Intervention:**

Laparoscopic myomectomy was performed.

**Outcomes:**

The total weight of the leiomyoma removed was 660 g with pathological diagnosis of uterine leiomyoma. The postoperative course was uneventful. The patient was free of disease at the follow-up consultation 18 months after the treatment.

**Lessons:**

Laparoscopic approach is a very useful and minimally invasive surgery for symptomatic leiomyoma in adolescents.

## 1 Introduction

Uterine leiomyomas are benign gynecological tumors originating from smooth muscle cells of the uterine wall. They are very common among woman of reproductive age, being found in an estimated 20% to 30% of women under 50 year of age.^[1–3]^ Many risk factors are associated with the development of leiomyomas, such as early menarche, obesity, and nulliparity with exposure to sex steroid hormones, especially estrogen.^[3,4]^ However, these lesions are extremely rare in adolescents, especially under 15 years of age, and the biological behavior of such leiomyomas is unknown, as is the best possible treatment. We herein report a uterine leiomyoma in a 13-year-old adolescent treated by laparoscopic myomectomy.

## 2 Case presentation

This study was approved by the Osaka Medical College Clinical Research Review Board, and the patient with her parents gave written informed consent for publication.

13-year-old girl with no gravida and her first menses at 11 years of age had a regular menstrual cycle with 4 to 5 days of bleeding each month. She had no remarkable medical history or family history. She had felt gradual abdominal distention over the past several months. Her body mass index was 18 kg/m^2^.

**Figure 1:**
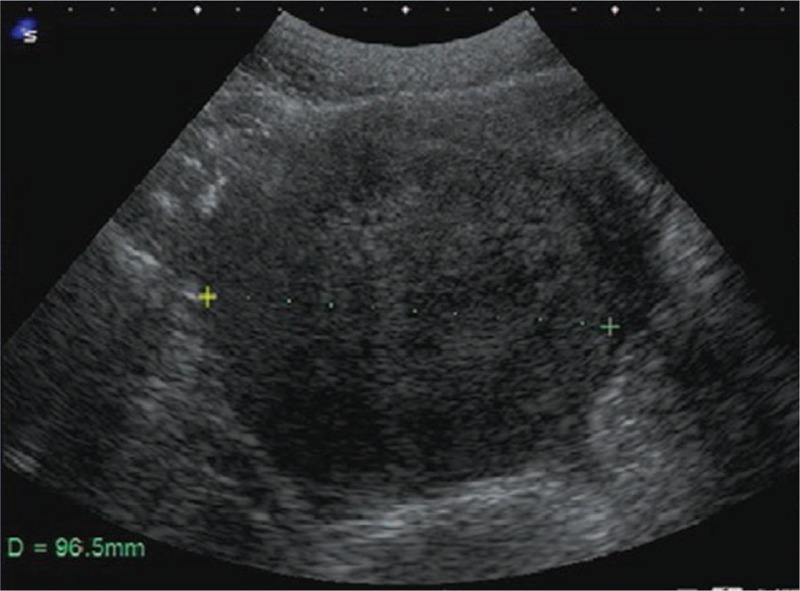
Transabdominal ultrasonographic image. There was a solid mass measuring 9.6 cm behind the uterus. The tubes and ovaries were not visualized because they were obscured by the enlarged uterus.

**Figure 2:**
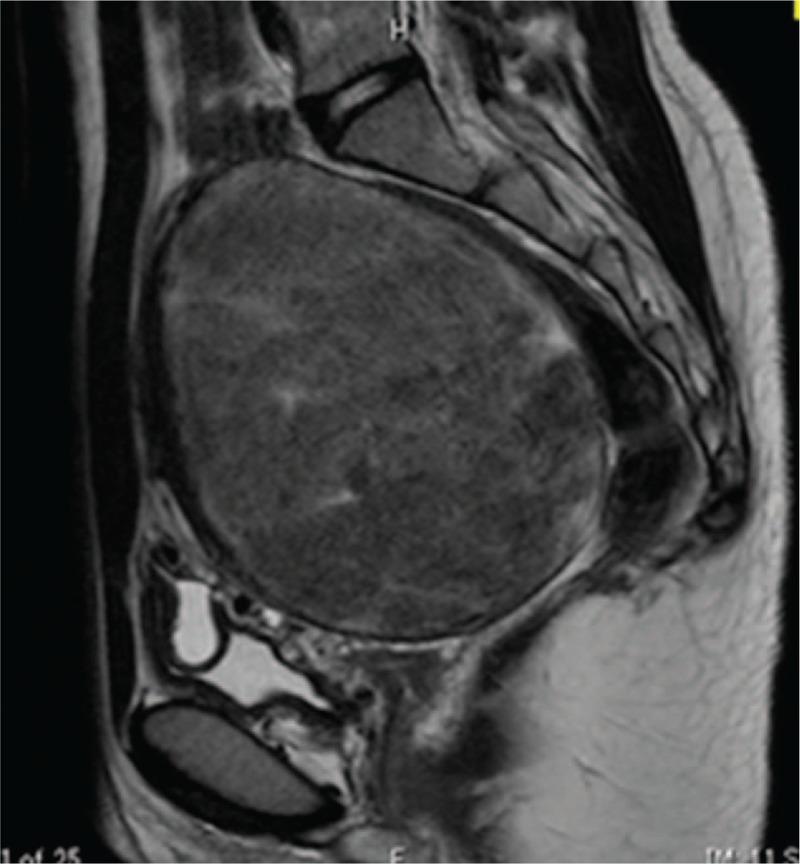
Magnetic resonance imaging (sagittal plane) revealed the solid mass to be within the uterine posterior wall, measuring 11 cm. There was no denaturation in the uterine tumor nor any suggestions of malignancy. It was suspected of being an intramural leiomyoma.

Transabdominal ultrasonography revealed a solid mass with a diameter of 10 cm located behind the uterus (Fig. 1). The findings of a blood examination, including tumor markers, were within normal limits. Magnetic resonance imaging (MRI) revealed the mass to be within the uterine posterior wall measuring 11 cm at presentation. There was no denaturation in the uterine tumor and no suggestions of malignancy (Fig. 2). Laparoscopic myomectomy was scheduled because of her abdominal distention.

**Figure 3:**
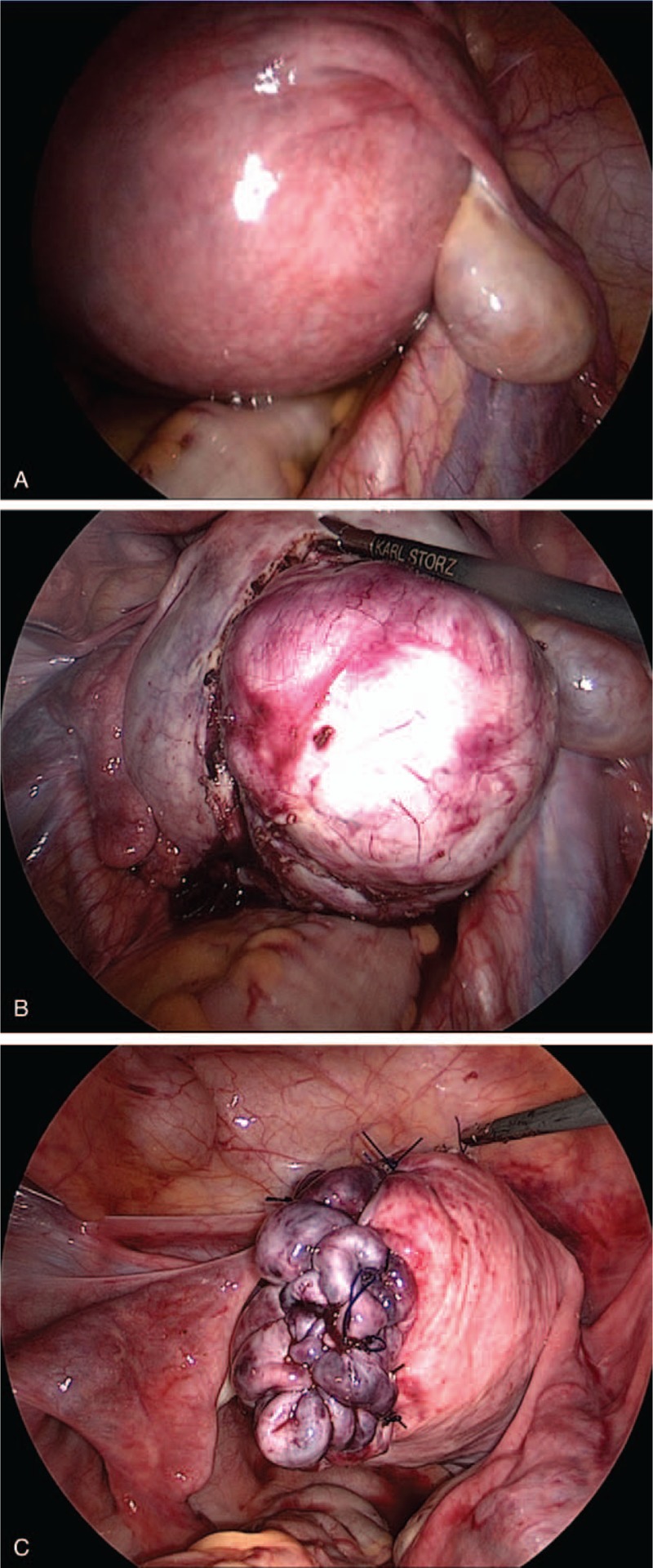
(A) Intraoperative laparoscopic image of the leiomyoma. The uterus was the size of a newborn's head with a large intramural leiomyoma. Both tubes and ovaries were normal. There was no abdominal adhesion. (B) The uterine myometrium was incised for myomectomy. (C) The myometrium with defection for myomectomy was sutured with 2-0 PDS.

Surgery was performed with peumoperitoneum under general anesthesia. She underwent multiport (4 ports) laparoscopic myomectomy with parallel procedures. The uterus itself was the size of a newborn's head with normal tubes and ovaries (Fig. 3A). There was no adhesion. A dilute pitressin was injected into the myometrium. The serosal surface of the leiomyoma was incised in a monopolar direction, and dissection was performed with care taken to avoid entering the uterine cavity (Fig. 3B). After myomectomy, the leiomyoma was cut into smaller pieces inside a plastic bag (MemoBag, Teleflex, Japan) and retrieved from abdominal cavity. The myometrium was closed a using a simple interrupted suture and a baseball suture with 2-0 PDS (Ethicon, Johnson & Johnson, Somerville, NJ, USA) (Fig. 3C). The total weight of the leiomyoma removed was 660 g, and the surgery lasted for 5 hours and 17 minutes with 1050 ml of blood lost (864 ml restored by Cell Saver; Haemonetics Co., Braintree, MA, USA).

The postoperative course was uneventful. Pathologically, the tumor was diagnosed as a uterine leiomyoma. Eighteen months have passed since the treatment, and no recurrence has been noted.

## 3 Discussion

In the current case, we performed laparoscopic myomectomy for a 13-year-old girl with uterine leiomyoma. To our knowledge, this is the youngest case in which laparoscopic myomectomy was performed.

Uterine leiomyomas are a very common gynecological tumor in woman of reproductive age; however, they are extremely rare in adolescents (1.0%).^[5]^ Clinically, a number of symptoms of leiomyomas in adolescents have been consistently reported in the literature. Moroni et al described the outcomes of 19 case reports of fibroids in adolescents from 1969 to 2014, with 87.5% (17/19) of patients presenting with symptoms, including abnormal uterine bleeding (10/18), abdominal pain (6/18), and perception of an abdominal mass or increased abdominal volume (8/18). Among the identified cases, the average age was 15.35 years, and the mean diameter was 12.28 cm. The most common treatment was abdominal myomectomy (13/19), with hysteroscopic myomectomy (2/19) and abdominal hysterectomy (1/18) also reported.^[5]^ There has only been one case report of leiomyoma in an adolescent successfully treated by laparoscopic myomectomy.^[6]^

Laparoscopic salpingo-oophorectomy for benign adnexal tumor, such as serous cystadenoma and dermoid cyst, in adolescents is not rare. Recent studies have shown no significant difference in surgery time, duration of hospitalization, blood loss, or postoperative complications between adolescents and adults.^[7,8]^ However, laparoscopic myomectomy may be difficult in adolescents for several reasons. In symptomatic adolescent cases, most leiomyomas are already large at the time of the diagnosis. In addition, the intraoperative space is restricted in adolescents due to their relatively small body size. The risk of complications, including blood loss and organs injury, may therefore be increased.

Parker et al found that up to 11% of women have a history of single fibroid removal and 26% have a history of multiple fibroid removal require secondary surgery for recurrence of leiomyoma. Laparoscopic and abdominal methods of myomectomy have similar recurrence rates.^[9]^ However, the incidence of postoperative pelvic adhesions is higher in abdominal myomectomy than in laparoscopic myomectomy.^[10]^ Therefore, laparoscopic myomectomy should be selected as the primary surgery of leiomyoma in order to create a safe environment for secondary surgery and reduce the risk of postoperative complications.

Gonadotropin-releasing hormone (GnRH) analogues cause the profound inhabitation of sex hormones, including estrogen and androgen, and are used for a variety of indications. GnRH analogues are particularly commonly prescribed for leiomyoma in adult woman to reduce the size of the mass and make surgery easier and safer.^[11]^ However, they have not been prescribed to this end in adolescents. The side effects of GnRH analogues in adolescents (as well as adults) must be considered, such as hot flashes, osteoporosis, dyslipidemia, hypertension, and the suppression of secondary sexual development. Bone loss leading to osteoporosis after long-term use is the most serious complication and may limit the therapy. A rule of thumb for women with endometriosis is that approximately 6% of bone is lost over 12 months of therapy, and 3% is regained following the cessation of therapy.^[12]^ Therefore, adolescents with early puberty or gender dysphoria are generally treated with GnRH analogues; however, whether this treatment should be started at an early Tanner stage or when puberty is almost complete and the appropriate duration of treatment remain unclear at present; such decisions may depend on the individual child and how well he or she is growing.^[13,14]^ GnRH analogues may be useful for reducing the size of the mass and making laparoscopic surgery easier and safer in adolescents as well as in adults. However, we advise against the use of these agents for leiomyoma in adolescents due to a lack of evidence.

It is difficult to define the impact of myomas in adolescent on future fertility. Five to 10% of infertile women have uterine myomas, however, it is unknown whether the myoma cause infertility directly. Many studies showed that myomectomy increased pregnancy rate and improve assisted reproductive technology success if the myomas are in or abutting the endometrial cavity or if they are large.^[15]^

In conclusion, laparoscopic myomectomy can be performed depending on the size, number, and location of the mass and the skill of the surgeons involved. This approach is a very useful and minimally invasive surgery for symptomatic leiomyoma in adolescents that reduces the postoperative pain, risk of pelvic adhesions, and hospitalization duration and affords a good cosmetic appearance.

## Acknowledgments

The authors thank Junko Hayashi and Kumiko Satoh for their valuable secretarial assistance.

## Author contributions

**Writing – original draft:** Natsuko Morita, Tomohito Tanaka.

**Writing – review & editing:** Tomohito Tanaka, Sosuke Hashida, Satoshi Tsunetoh, Kohei Taniguchi, Kazumasa Komura, Masahide Ohmichi.
